# A Bayesian Approach to Latent Class Modeling for Estimating the Prevalence of Schizophrenia Using Administrative Databases

**DOI:** 10.3389/fpsyt.2015.00099

**Published:** 2015-07-06

**Authors:** Vincent Laliberté, Lawrence Joseph, Ian Gold

**Affiliations:** ^1^Department of Psychiatry, McGill University, Montreal, QC, Canada; ^2^Department of Epidemiology and Biostatistics, McGill University, Montreal, QC, Canada; ^3^Department of Philosophy, McGill University, Montreal, QC, Canada

**Keywords:** psychiatry, schizophrenia, epidemiology, urbanicity, psychosis, administrative data, environment

## Abstract

Estimating the incidence and the prevalence of psychotic disorders in the province of Quebec has been the object of some interest in recent years as a contribution to the epidemiological study of the causes of psychotic disorders being carried out primarily in UK and Scandinavia. A number of studies have used administrative data from the *Régie de l’assurance maladie du Québec* (RAMQ) that includes nearly all Quebec citizens to obtain geographical and temporal prevalence estimates for the illness. However, there has been no investigation of the validity of RAMQ diagnoses for psychotic disorders, and without a measure of the sensitivity and the specificity of these diagnoses, it is impossible to be confident in the accuracy of the estimates obtained. This paper proposes the use of latent class analysis to ascertain the validity of a diagnosis of schizophrenia using RAMQ data.

## Introduction

The link between urban living and psychosis has intrigued researchers since the first description of the phenomenon in the seminal studies of Faris and Dunham (1, 2) who found higher rates of schizophrenia in Chicago neighborhoods characterized by social isolation and disorganization but found no parallel difference for bipolar disorder. Since then, a large number of studies have confirmed the relationship between city size and schizophrenia as well as a dose–response association (3–6). The correlation remains unchanged whether schizophrenia or psychosis, more broadly, is under consideration (6) and holds true both of the relation between psychosis and absolute city size and population density. A meta-analysis by Vassos et al. (6) found that relative to the most rural environment, the most urban environment is associated with a 2.37-fold risk of schizophrenia (6).

The majority of studies on the urban effect in schizophrenia have been carried out either in large metropolises [particularly London (7), with a population of approximately 8.3 million] or the relatively small cities of Scandinavia [especially Copenhagen (8), with a population of approximately 0.6 million]. Little data exist for urban areas intermediate between these. Our purpose here is to outline an approach to studying the prevalence of schizophrenia in the Canadian province of Quebec with a particular focus on Montreal. An island city with a population of approximately 1.89 million (9), Montreal is the largest urban region in Quebec, and a French-speaking enclave in North America. In addition to its linguistic diversity, Montreal is also a hub for new immigrants to Canada and is thus home to a great variety of cultural and ethnic communities. In 2005, in the neighborhood of Côte-des-Neiges, one of the most diverse neighborhoods in Canada, some 51% of the 100,000 residents were immigrants (10). Unlike some more homogeneous North American cities, Montreal is also marked by significant social differences across neighborhoods. For these reasons, Montreal presents a particularly interesting laboratory in which to explore the social effects of urban living on schizophrenia. Interestingly, a study conducted in 2013 showed that the incidence rate of the disorder was higher in the Island of Montreal than in the next-largest urban area in the province, Quebec City (11).

The first part of this paper examines how administrative databases have been used in Quebec and other provinces of Canada to study the prevalence and incidence of psychotic disorders. (Prevalence measures the number of cases of a disease at a single moment in time, while incidence measures the risk for a single individual of developing a disease during a given period.) We pay close attention to the question of the validity of the diagnosis recorded in the database. Although this question has been addressed in the context of a number of diseases and conditions, it has not been examined in the case of schizophrenia or other psychotic disorders. We then describe an ascertainment methodology based on Bayesian latent class modeling that makes it possible to estimate the validity of the diagnosis of schizophrenia. Finally, using previously published data, we show that the new method delivers results that are significantly different from those currently available.

## The Urban Effect

The causes of the urban effect in schizophrenia are currently unknown. However, it represents an important problem for the theory of schizophrenia, because there is little evidence that urban living contributes to an increased risk of schizophrenia by means of straightforward biological mechanisms. For example, the urban effect does not seem to be a function of increased viral transmission, the increased availability of drugs of abuse, or the tendency of immigrants – also at increased risk of schizophrenia – to move to cities. Further, the evidence is incompatible with the hypothesis that people with schizophrenia merely tend to drift to urban areas – the so-called “social drift” hypothesis [(4, 12–15); see also Ref. (16)].

In contrast, there is evidence that the risk of urban living on schizophrenia is raised as a function of social processes. Some of the most compelling evidence for this view comes from the discovery of differential rates of schizophrenia across neighborhoods of London. It is assumed that if the urban effect in schizophrenia involves physical processes, one would expect the same prevalence across the city. Neighborhood differences, however, are social differences (7, 17, 18).

The best known theoretical proposal concerning how social factors might contribute to an increased risk for schizophrenia is the “social defeat” hypothesis of Selten and Cantor-Graae [(19); see also Ref. (20)] according to which experiences of subordination or humiliation have effects on the hypothalamic–pituitary–adrenal axis, which alter dopamine function and thereby alter the risk of psychosis (21). Since experiences of subordination are more common among individuals in ethnic or other minority groups, a consequence of this view is that discrimination may be an indirect cause of schizophrenia. This hypothesis is supported by the finding that the risk of developing psychotic symptoms increases with perceived discrimination (22).

A second possibility – not incompatible with the first – is that “social fragmentation” may be the most important factor explaining the higher rate of schizophrenia in urban settings (23). This fragmentation could manifest at the level of family structure (12, 24) or may be a property of whole neighborhoods (25). Indeed, a study by Kirkbride and colleagues measured voter turnout in council elections as a marker of social cohesion (i.e., low social fragmentation) and found lower rates of schizophrenia in less fragmented neighborhoods (7).

## Epidemiological Studies of Psychotic Disorders in the Province of Quebec

Because epidemiological studies of disease prevalence can be prohibitively expensive and labor intensive, estimating prevalence usually depends on the availability of administrative data – that is, data collected (typically by the government) for administrative purposes (26). In Quebec, the medical system is almost exclusively public, and diagnostic and treatment data are collected for insurance purposes by the *Régie de l’assurance maladie du Québec* (Quebec Office of Health Insurance; RAMQ). The RAMQ was established in 1969 to provide Quebec residents with universal health insurance covering physician as well as hospital services, in addition to managing health administration databases. The data are collected routinely in order to compensate physicians and hospitals as well as to monitor or manage programs. Since medical data are linked to precise geographic information available for research purposes, prevalence rates for neighborhoods can be inferred. But because RAMQ data are not collected for research purposes, but rather for the purpose of physician reimbursement, the primary challenge to using it is establishing its reliability (27). There is considerable anecdotal evidence of inaccuracy in the diagnosis indicated on administrative forms, which may be attributable to the physician, or to the many individuals involved with the recording of the information (28). In addition, in the absence of any biomarkers, different clinicians may disagree on a given diagnosis, and because professionals do no get paid by diagnosis, the incentive to get it right is not as great as it might otherwise be. Moreover, little research has been carried out into the validity of psychiatric diagnosis included in the RAMQ database (29, 30).

Byrne et al. (31) address the issue of validity in psychiatric studies based on administrative registers in a systematic review of 14 papers, most of which are concerned with schizophrenia. The authors conclude that there is a general lack of clarity about what counts as a valid diagnosis and how to measure it (31). Indeed, Rawson et al. (28) point out that even though the potential for biases are frequently acknowledged, administrative data have been uncritically accepted as valid and reliable (28). There is, therefore, a need to properly evaluate the results of epidemiological studies of schizophrenia using administrative data, not least because the results are used by health planners and policy makers to develop and implement intervention services (11). More importantly, for present purposes, a validation of RAMQ data on schizophrenia is necessary in order to carry out the finer-grained analysis required to better understand the social factors at play in the genesis of the illness. A more precise method for assessing the prevalence of schizophrenia in Quebec would make it possible to study subtle variations across and within neighborhoods.

The use of administrative data for epidemiological studies is not new; it has been used in Quebec (32) and elsewhere (33) to study various diseases including schizophrenia (34). RAMQ data, in particular, have been used to estimate the prevalence and incidence of various diseases (35–37), as well as different forms of psychotic disorder in Quebec (11, 29, 30). The main advantages of using RAMQ databases are ease of access and low cost (29). Because continuous data are available from 1980s to 1990s (depending on the particular dataset) (38), longitudinal investigations are possible (29). In addition, data are exhaustive since there are no privately funded facilities where patients with schizophrenia receive treatment (30).

The RAMQ collects information through various sources (38). The “Quebec physician claims” database includes fee-for-service physician claims for services occurring in a hospital, such as a physician visit or consultation, a surgery, or post-operative care. It also includes claims for ambulatory (out-of-hospital) care – for example, physician visits in a doctor’s office, an emergency room visit, or a minor surgery (39). The physician claim includes a diagnosis based on the international classification of diseases (ICD), the physician specialty, as well as codes indicating medical procedures to be carried out based on the Canadian Classification of Diagnostic, Therapeutic, and Surgical Procedures, and the location of the procedure. The MED-ECHO data source provides the discharge summary following any hospitalizations, such as acute care, long-term care, and day surgery, and includes diagnosis based on the same classification. A primary diagnosis is provided, as well as up to 19 secondary diagnoses. Records are also available from frontline public health and social services clinics (*Centres local de services communautaires; CLSCs*) as well as residential and long-term care facilities (*Centres d’hébergement et de soins de longue durée; CHSLDs*). Sociodemographic information, such as age and sex, the location of the residence of the patient based on a six-digit postal code, and a unique personal identification number are included. Indicators of social and material deprivation are also collected (30). Finally, the RAMQ collects data on drugs prescribed as well as their dosages (30).

Three epidemiologic studies have been carried out on psychotic disorders using RAMQ data. In the first, Anderson et al. (30) estimated the incidence of first-episode psychosis in Montreal (30). To be included in the study, patients had to be between 14 and 25 years old and have resided in Montreal between 2004 and 2006. Cases were associated with one of the following: (a) a physician claim for schizophrenia-spectrum psychosis (SSP), which includes schizophrenia, schizophreniform disorder, schizoaffective disorder, or delusional disorder, with a psychiatric procedure; (b) a local community clinic visit for schizophrenia or other psychotic disorder and a procedure code for a mental health or emergency visit; or (c) a hospitalization with a primary or secondary discharge diagnosis of SSP. Patients with a previous medical claim for a psychotic disorder or a previous prescription of antipsychotic medication were excluded. The authors identified 456 patients diagnosed with a SSP, and after adjusting for age and sex, they estimated that there were 82.9 cases per 100,000 men per year, and 32.2 cases per 100,000 women per year. The diagnosis of psychotic disorder was made by a psychiatrist in 69% of cases, and in 60% of cases the diagnosis was established in the emergency room. Anderson et al. (30) also made use of two indices of social and material deprivation as an estimate of socioeconomic disparity, which have a high level of geographic resolution (40). The authors found that the most deprived areas had a higher incidence of SSP.

The objective of the second study of Vanasse et al. (29) was to develop a method to estimate both the prevalence and the incidence of the diagnosis of schizophrenia by comparing four different case selection algorithms (29). Data were collected in Quebec’s physician claims as well as in the hospital discharge (MED-ECHO) databases. Every adult given a hospital discharge with a diagnosis of schizophrenia or associated with a physician claim for it from January 1996 to December 2006 was included in the analysis. The algorithms used either physician claims alone, or a mix of hospital and physician claims. Algorithm 1 is based only on hospitalization with a diagnosis of schizophrenia; algorithm 2 on either a hospitalization or an emergency room visit with a diagnosis of schizophrenia; algorithm 3 takes into account either a hospitalization or a visit to a psychiatrist with a recorded diagnosis of schizophrenia; and algorithm 4 is based on either a hospitalization or a visit to any physician with a recorded diagnosis of schizophrenia. The lifetime treatment prevalence of schizophrenia (i.e., the percentage of the population who received a diagnosis of schizophrenia during 11 years period of the study) was estimated to vary from 0.59 to 1.46% and the incidence was estimated to be between 42 and 94 cases per 100,000 individuals in the year 2006. In a subsequent paper, this research team studied the incidence of schizophrenia between 2004 and 2007 (11). Patients included in the study were 18 years of age or older and had received a diagnosis of schizophrenia recorded in one of the same two databases used in the first study. The incidence rate of cases was found to be higher in the urban area Montreal than in other regions (11).

Other estimates of the prevalence of schizophrenia have been conducted in Canada using administrative databases. A Canadian study used administrative data in British Columbia and estimated the prevalence of schizophrenic disorders to vary from 0.42 to 0.45 cases per 100,000 (34). A report from the *Agence de la santé et des services sociaux de Montréal* (Montreal Health and Social Services Agency), which comprises 18 regional authorities supporting the Ministry of Health and Social Services (41), estimated the 1-year prevalence of schizophrenia in Montreal to be 0.57% (42).

## A Limitation of Epidemiological Studies of Psychosis Based on Administrative Databases

An important limitation of these studies is their presumption that the diagnosis of schizophrenia as found in the administrative database is valid. That is, all of the studies assume that the method of ascertainment used is both 100% specific and 100% sensitive. In the absence of a proper estimation of the validity of the diagnosis under investigation, however, relying on these results requires a leap of faith. The problem is particularly acute given the fact that the primary purpose of RAMQ data is physician reimbursement. As we note above, a systematic review of psychiatric studies based on administrative data finds that there is considerable lack of clarity about how to validate diagnoses (31).

There is some circumstantial evidence for the accuracy of psychiatric diagnoses as found in administrative databases. A 2009 study, for example, showed that administrative data in Canada could be reliably used for mental disorder surveillance (43) (i.e., the continuous collection of data to plan and implement mental health policy) (44). Moreover, an estimate of the prevalence of bipolar disorder based on administrative data in Canada was found to be comparable to the results of a health survey (45). However, mental health surveys may underestimate the real burden of mental disorders due to fear of stigmatization or selection bias (29), and, more generally, because of inaccuracies in self-reports of mental illness.

A study of the validity of a diagnosis for schizophrenia and depressive disorder from a Canadian administrative database in Saskatchewan was conducted by Rawson et al. (28). In particular, they compared diagnoses from three sources of data and reported the percentage of agreement between each pair. The concordance was found to be 94% between computerized hospital data and medical charts for schizophrenia. Without an attempt to estimate specificity by including a random sample of non-schizophrenic subjects, however, the validity analysis remains incomplete. Interestingly, a study designed to validate the diagnosis of schizophrenia as found in the Swedish National Inpatient Register (46) found that the proportion of “true” cases was high and the number of false positives was no different in the hospitals in larger cities than those in smaller towns (46).

In contrast, however, there is a wealth of evidence from epidemiological research into other diseases to suggest that the assumption of perfect sensitivity and specificity of the diagnostic codes in a database does not hold (47). A satisfactory validation of the diagnosis of schizophrenia in the RAMQ database is required, therefore, before one can be confident of using this data for epidemiological purposes.

## A Bayesian Latent Class Model for Prevalence Estimation

To produce an adjusted prevalence estimate, one needs a good estimate of the sensitivity and specificity of the diagnoses in the database. For example, if *P* is the observed proportion of positive diagnoses in the database, π is the true prevalence, and *S* and *C* are the sensitivity and specificity, respectively, then we can reason as follows: positive results can arise either by the test correctly detecting a truly positive case, or by the test falsely detecting a truly negative case. The first case will arise with probability π*S, since the case is positive with probability given by the prevalence π, and the test will correctly detect a true positive with probability given by the sensitivity, *S*. The second case will arise with probability (1−π)*(1−*C*), since the case is negative with probability given by one minus the prevalence, or (1−π), and the test will incorrectly label a true negative as positive with probability given by one minus the specificity, or (1−*C*). Therefore, the total probability of the test coming up positive is given by

P=π*S+(1−π)(1−C),

and solving this algebraic equation for the prevalence π gives

π=(P+C−1)/(S+C−1).

Note that if *S* = *C* = 1 (sensitivity = specificity = 100%) then we are back to the standard assumption of perfect accuracy, and the estimate of the prevalence reduces to the rate of positive tests, *P*. Otherwise, the prevalence will be different from the observed “prevalence” as given by the number of positive tests, and in many cases the true prevalence can be very far from *P*.

There are at least two ways to establish the sensitivity and specificity of diagnostic codes or other clues found in administrative databases for any disease or condition. The most direct method is to gather a large enough sample of known cases and controls, and check for the presence or absence of the diagnostic code in the database. The rate of positive diagnoses found in the database among truly positive subjects is an estimate of the sensitivity, and the rate of negative diagnoses found among truly negative subjects is an estimate of the specificity. Such studies can be time consuming, but are feasible to carry out provided there is a source of known cases and controls.

Alternatively, one can use latent class models (48, 49), which can be divided into two types, identifiable and non-identifiable. Roughly speaking, identifiable latent class models are those where all unknown parameters can be estimated without the use of information external to the data set. One example would be when three tests or “clues” in the database are available. For example, in addition to the main diagnostic code, one can look for the presence or absence of medications and medical procedures that would be particular to the disease or condition under study. If three independent clues can be found, then there are seven parameters (sensitivity and specificity for each of the three clues along with the prevalence) to estimate from the 2 × 2 × 2 table of data, which provides seven degrees of freedom. Thus, one can use standard frequentist latent class models (50) or a Bayesian approach with minimum information priors (49) to estimate all parameters simultaneously. The Bayesian approach uses the same model (likelihood function) as the frequentist approach, the only difference being the prior information input by the Bayesian method. However, if minimum prior information is input (through what is often loosely termed as non-informative priors) then the two methods will provide numerically similar inferences.

Non-identifiable latent class models are used when there are more parameters to estimate than there are degrees of freedom. Such a model can be used, for example, when only the main diagnostic code is used to ascertain case status. In that situation, there will be three parameters to estimate (sensitivity and specificity of the diagnostic code, and the prevalence), but only one degree of freedom in the yes/no diagnostic data. In this case, one must employ information from outside the data set in order to provide reasonable inferences, with the information ideally coming from a prior study that outlined above to estimate the sensitivity and specificity of the diagnostic code, following which one can estimate the prevalence. Here, only Bayesian analysis can provide reasonable inferences (48).

Of course, it will not be possible to assess the validity of every diagnosis of psychotic disorders; it will be necessary to choose a sub-population. For this population, we would need to compare the diagnosis as found in the database to another measure, such as the hospital chart, which would be used as a proxy measure for the true presence of the illness. Hospital charts might sometimes be wrong due to misdiagnosis, and some individuals might never be treated in the health care system and so fail to be represented as having schizophrenia in the data set. In this case, we return to a non-identifiable model but with one of the “tests” presumably providing high enough sensitivity and specificity to lead to accurate prevalence estimates, using the prevalence conversion formula given above.

Our study will proceed in three stages. First, we will estimate the sensitivity and specificity of RAMQ-based diagnoses of schizophrenia by collecting a sample of known cases with and without schizophrenia, and comparing RAMQ diagnoses to hospital charts. These cases will come from the Psychotic Disorder Programs offering services to patients aged from 18 to 65 suffering schizophrenia and other forms of psychosis in the catchment area covered by the Douglas Mental Health University Institute (51) in Montreal, while controls will be individuals randomly selected in the same area. The Douglas Institute represents the highest level of care for schizophrenia in Montreal and will thus provide a best case scenario for estimating sensitivity.

In the second stage, we will use RAMQ data to estimate the prevalence of schizophrenia, adjusting by the sensitivity and specificity estimates obtained from stage 1. Note that the sensitivity and specificity estimates here will be entered as prior densities, and not point estimates, since even with the most carefully designed first step, these quantities will not be known exactly. The probability densities will thus reflect the uncertainty associated with not knowing the exact values for these quantities.

In the third stage, we will re-estimate the prevalence of schizophrenia using three tests from the RAMQ database. One test will be the imperfect diagnosis as recorded in the database. The second test will be the prescription of antipsychotic medications at a therapeutic dosage for schizophrenia, and the third will be hospitalizations or a psychiatric procedure code. With these tests, we can estimate sensitivity and specificity by means of latent class modeling. We will thus be able to check the robustness of the prevalence estimate from step 2, as well as re-estimate and compare with the estimates of the sensitivity and specificity from step 1.

Finally, since we are interested in comparing the rates of schizophrenia in different districts of Montreal, we will repeat the above steps for each sub-area of interest. This can be done by running separate latent class models within each of the areas of interest, and also via one larger hierarchical Bayesian model that incorporates a latent class component. Separate models have the advantage of independence of estimates, but may suffer from small sample sizes. On the other hand, hierarchical models pool all data together, and hence, use a larger sample size, but represent a trade-off between bias and precision. We will run both methods and compare the robustness of the results across methods.

Since the publication of the seminal paper on the Bayesian estimation of disease prevalence (51), this methodology has been used in a wide array of epidemiological studies and its validity has been well recognized. However, to our knowledge, it is the first time that it will be used for the estimation of the prevalence of a psychiatric disorder using an administrative database.

## Application of the Bayesian Latent Class Model to Previous Studies

As an illustration of an application of our method consider the result of Vanasse et al. (29) discussed above. They estimated a prevalence of schizophrenia in Quebec of 0.59–1.46% using algorithms assuming 100% sensitivity and specificity in order to derive their estimates. The sensitivities and specificities from each algorithm are unknown, but are surely <100%. Let’s suppose we assume imperfect – but still excellent – sensitivity and specificity values of 99.5, and 1% prevalence, which corresponds roughly to the mid-point of their range. Using these values, we derive an adjusted prevalence of 0.5%, which is lower than even their lower limit. Clearly, small deviations for the assumption of perfect sensitivity and specificity may lead to substantial errors in prevalence estimation.

A further demonstration of the power of the technique when applied to RAMQ data is contained in Ladouceur et al. (49). In that paper, an unadjusted osteoarthristis prevalence estimate of 10.0% was adjusted to 14.8%, an increase of almost 50%. Thus, unadjusted estimates from administrative databases must be treated with caution.

## Conclusion

The Quebec health care system was not created for the purpose of conducting epidemiological studies, but the administrative data available through RAMQ provide a valuable opportunity to conduct epidemiological research. Since there are good reasons to believe that schizophrenia is linked to the social world, the use of administrative data makes it possible to get access to a large database of information that can be linked to social variables in a much less labor-intensive fashion than would otherwise be necessary. This effort will only bear fruit, however, if we can have confidence in the estimates it delivers; the Bayesian approach sketched above will go some way to providing that confidence. A more precise method for assessing the prevalence of schizophrenia in Quebec would make it possible to carry out the finer-grained analysis required to better understand the social factors at play in the genesis of the illness – in particular, the social variations that correlate with illness across and within neighborhoods. With this data in hand, the picture of schizophrenia in Quebec will fill a significant gap in the epidemiological study of schizophrenia.

## Author Contributions

VL designed the work and elaborated the specifics of the study. He drafted the work and revised it. He also took charge of the correspondence with editors. LJ gave a significant ­contribution in designing the work and the specifics of the study. He also significantly contributed to elaborating the methodology exposed in the article. LJ drafted the work and revised it critically for important intellectual content. IG gave a significant contribution in designing the work and elaborating the ­specifics of the study. He revised the work critically for important intellectual content. The three authors (Vl, LJ, and IC) worked toward final approval of the work and agree to be accountable for all its aspects.

## Conflict of Interest Statement

The authors declare that the research was conducted in the absence of any commercial or financial relationships that could be construed as a potential conflict of interest.
